# Effect of α-glycosidase inhibitors from endophytic fungus *Alternaria destruens* on survival and development of insect pest *Spodoptera litura* Fab. and fungal phytopathogens

**DOI:** 10.1038/s41598-019-47838-6

**Published:** 2019-08-06

**Authors:** Jasleen Kaur, Avinash Sharma, Manish Sharma, Rajesh Kumari Manhas, Sanehdeep Kaur, Amarjeet Kaur

**Affiliations:** 1 Department of Microbiology, Guru Nanak Dev University, Amritsar, 143005 India; 2 Department of Zoology, Guru Nanak Dev University, Amritsar, 143005 India

**Keywords:** Fungal host response, Environmental microbiology

## Abstract

In the present study the production of α-glycosidase inhibitors was used as a strategy to screen endophytic fungi with insecticidal and antifungal potential. Endophytic fungi were isolated from *Calotropis gigantea* L. (Gentianales: Apocynaceae) and evaluated for their α-glycosidase inhibitory activity. Maximum inhibitory activity was observed in an isolate AKL-3, identified to be *Alternaria destruens* E.G.Simmons on the basis of morphological and molecular analysis. Production of inhibitory metabolites was carried out on malt extract and partially purified using column chromatography. Insecticidal potential was examined on *Spodoptera litura* Fab. (Lepidoptera: Noctudiae). Partially purified α-glycosidase inhibitors induced high mortality, delayed the development period as well as affected the adult emergence and induced adult deformities. Nutritional analysis revealed the toxic and antifeedant effect of AKL-3 inhibitors on various food utilization parameters of *S*. *litura*. They also inhibited the *in vivo* digestive enzymes activity in *S*. *litura*. Partially purified α-glycosidase inhibitors were also studied for their antifungal potential. Inhibitors demonstrated antifungal activity against the tested phytopathogens inducing severe morphological changes in mycelium and spores. This is the first report on production of α-glycosidase inhibitors from *A*. *destruens* with insecticidal and antifungal activity. The study also highlights the importance of endophytes in providing protection against insect pests and pathogens to the host.

## Introduction

Insect pests and fungal pathogens have become a source of concern as great losses to economically important crops are caused by them worldwide^1–4^. To alleviate their effect, excessive dependence on chemical pesticides has resulted in many environmental issues such as contamination of food and water sources, poisoning of non-target beneficial insects and resistance development in insect pests^5,6^. Researchers have focused on alternative methods to control pests with special emphasis on bio-control. Among bio-control agents, endophytes would be an ideal choice as these are integrated into the host plant. They are the microorganisms which spend the whole or part of their life cycle colonizing inter- or intra-cellularly, in the healthy living tissues of the host, without causing any symptoms^7^. These microbes confer benefits to the host plants such as greater access to the nutrients^8–10^, protection from insect pests, parasitic fungi, etc. and from abiotic stresses like desiccation^11,12^. The protective role of endophytes against insect pests and fungal pathogens has been well documented in literature^13,14^. Plants artificially inoculated with endophytic fungi were also found to demonstrate resistance against insect pests^15,16^. These microorganisms synthesize a number of bioactive compounds mainly alkaloids, phenols, terpenoids, sterols etc.^17^ which play a significant role in protecting their host plants from herbivores^18–20^. One of the mechanisms employed by these microbes for providing protection could be through inhibition of vital enzymes of pests and pathogens.

α-Amylase (3.2.1.1) and α-glucosidase (3.2.1.20) are glycoside hydrolase enzymes responsible for digestion of carbohydrates. These enzymes hydrolyze carbohydrates by acting on 1,4-α linkages, releasing D-glucose from the non-reducing end of the sugar^21^. In insects, these enzymes are found in the salivary secretions, hemolymph and alimentary canal^22^. The inhibition of these enzymes would slow the process of digestion; leading to adverse effects in the insects. The production of α-glycosidase inhibitors (AGIs) has been reported as an inherent mechanism in plants to resist herbivory and insect pests^23–26^. Transgenic plants expressing AGI genes have been produced and found to possess resistance against various insects^27–29^. Though, digestive enzyme inhibitors from plants have been extensively reported for their insecticidal activity^23–26^, there are few reports available of similar studies on bio control efficiency of enzyme inhibitors from endophytes. In this study, we evaluated the insecticidal potential of AGIs obtained from endophytic fungi against *Spodoptera litura* (Fab.). *S*. *litura* is a polyophagous lepidopteran pest causing huge ecomomic losses to variety of agriculturally important crops. Moreover, it has developed resistance to a number of commercially available insecticides^6^.

α-Glycosidase enzymes are also involved in processes during fungal growth and have a role in synthesis and extension of cell wall^30^. Inhibitors of such enzymes could affect the growth and development of fungi leading to antifungal activity. Keeping this in view, AGI potential of endophytic fungi isolated from *Calotropis gigantea* L. was used as a strategy for isolating potential strains with insecticidal and antifungal activity. Endophytes are known to produce compounds with similar properties as that of host plant through genetic recombination and vice versa^31–33^. *C*. *gigantea* was selected as it possesses, antifungal, antidiabetic and insecticidal potential^34–36^ and their endophytes might produce metabolites with α-glycosidase inhibitory activity.

## Results

**Figure 1:**
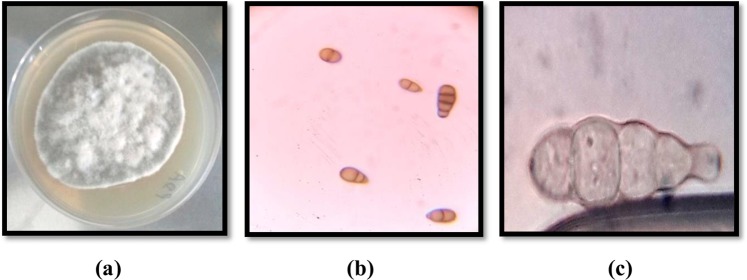
Morphology of (**a**) colony and (**b**,**c**) conidia at 40X and 100X of *A*. *destruens* AKL-3, respectively.

**Figure 2:**
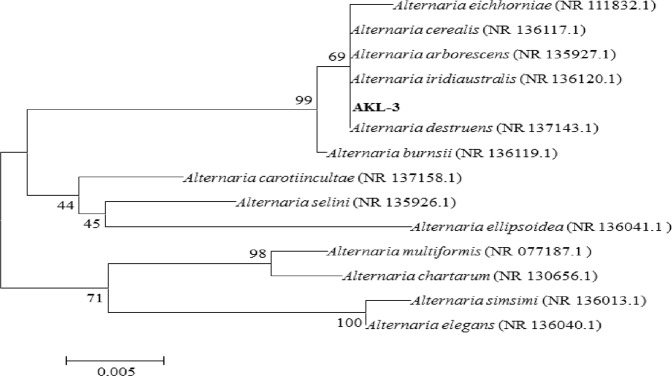
Phylogenetic tree showing the position of AKL-3 on the basis of ITS1-5.8 rDNA-ITS2 gene sequence. The evolutionary history was inferred using the Neighbor-Joining method. The analysis involved 14 nucleotide sequences. All positions with less than 95% site coverage were eliminated. There were a total of 461 positions in the final dataset. Evolutionary analyses were conducted in MEGA 6.

In the present study, 22 endophytic fungi were isolated from *C*. *gigantea* and screened for inhibitory activity against α-glucosidase and α-amylase. Six cultures exhibited α-glucosidase inhibitory activity in the range of 55–93.4% with maximum being found in AKL-3 (93.4%) followed by AKL-9 (84.4%). AKL-3 also inhibited α-amylase to the extent of 32% while other cultures did not show inhibition against α-amylase. Culture AKL-3 was selected for further studies and identified according to standard taxonomic key including colony diameter, color and morphology of hyphae and conidia. The colonies were slow growing having a diameter of 5.3 cm when incubated on Potato Dextrose Agar (PDA) plates at 30 °C for 9 d. These were white in color when young and turned greenish on maturity with dark reverse (Fig. 1a). Hyphae were septate and branched in the apical region, conidia were multi-celled with transverse as well as longitudinal septa and round to oval in shape. Longitudinal septa were fewer in number than transverse septa (Fig. 1b,c). The genetic relationship of AKL-3 was determined by amplification of ITS1-5.8S-ITS2 rDNA region. The size of the amplified sequence was 476 bp. After sequencing, the sequence was deposited with GenBank under accession number MH071380. Alignment with homologous nucleotide sequences, revealed the strain AKL-3 to be closest to *Alternaria destruens* with a similarity of 100% with type specimen (Fig. 2). Thus, on the basis of molecular and morphological analysis, the strain AKL-3 could be identified as *A*. *destruens*. The culture was submitted to National Centre for Microbial Resource (NCMR) at National Centre for Cell Science (NCCS), Pune, Maharashtra, India under accession number MCC1666.

Column chromatography of ethyl acetate extract of *A*. *destruens* AKL-3 yielded two active fractions (AF1 and AF2) which differed with respect to their color and also exhibited different TLC profiles. AF1 was yellow in color whereas AF2 was red. Active fraction AF1 inhibited α-glucosidase enzyme to an extent of 87.75% whereas AF2 showed 72.11% inhibition. Active fractions AF1 and AF2 were also assayed for their inhibitory potential against α-amylase and β-glucosidase. It was observed that AF1 was highly specific as it possessed α-glucosidase inhibitory potential but showed no inhibition against the other two enzymes (α-amylase and β-glucosidase), while active fraction AF2 exhibited inhibition against β-glucosidase (54.62%) as well as α-amylase (34.55%). Both the active fractions were found to possess phenolic compounds after staining with Fast Blue B and FeCl_3_.

### Insecticidal activity

Preliminary studies to determine the insecticidal potential were carried out on second instar larvae of *S*. *litura* by feeding them on artificial diet supplemented with 1.5 mg/ml of AF1, AF2 and after pooling them together. The mean average larval mortality recorded was 19.99, 23.33 and 33.3 percent due to AF1, AF2 and pooled fraction, respectively. The effect of pooled fraction was more evident than the individual fractions therefore detailed studies on various parameters *viz*. larval mortality, larval period, pupal period and total development period were conducted using different concentrations (0.5–2.5 mg/ml) of pooled fraction.

**Figure 3:**
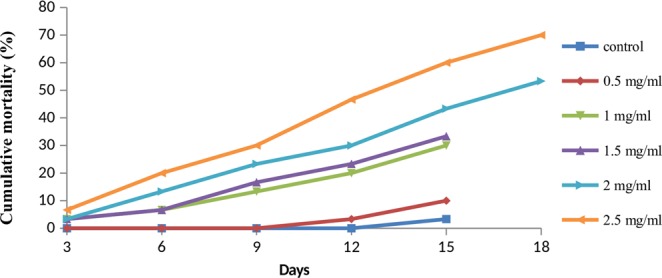
Mean cumulative mortality of second instar larvae of *S*. *litura* fed on diet supplemented with different concentrations of α-glycosidase inhibitors from *A*. *destruens* AKL-3.

**Figure 4:**
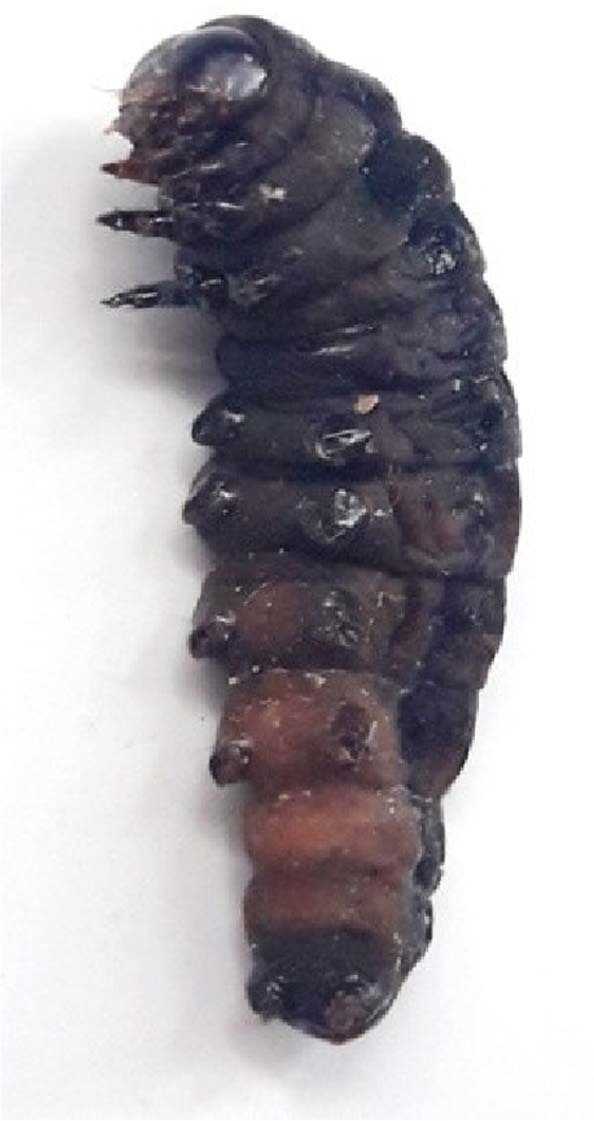
Failure of molting in larva of *S*. *litura* due to α-glycosidase inhibitors from *A*. *destruens* AKL-3.

Larval exposure to diet amended with varying concentrations of pooled fraction of *A*. *destruens* resulted in total average mortality of 10 to 70 percent as compared to 3.33 percent in control. The larval mortality increased in a dose dependent manner with significant effect at 2.0 and 2.5 mg/ml (F = 13.55, p ≤ 0.001). The mortality rate increased steadily with the increase in feeding duration (Fig. 3). The LC_50_ value was determined to be 1.875 mg/ml using probit analysis. Sluggishness and failure of molting were observed prior to larval deaths (Fig. 4). The negative impact of the inhibitors was also observed on growth and developmental parameters of *S*. *litura*. Relative to control, the larval period extended significantly by 3.64 and 4.55 days due to 2.0 and 2.5 mg/ml of pooled fraction of *A*. *destruens* respectively (F = 41.92, p ≤ 0.001; Table 1). Similarly, the pupal period was also prolonged with significant effect at these two higher concentrations (F = 3.45, p ≤ 0.001). In comparison to control, the development period of *S*. *litura* was delayed by 7.89 and 9.19 days when the larvae consumed 2.0 and 2.5 mg/ml of partial purified inhibitor as evident in Table 1. Toxic effects of pooled fraction were also detected on adult emergence as 23.33 percent adults emerged at the highest concentration as compared to 96.67 percent in control.

**Table 1:** Effect of α-glycosidase inhibitors from *A*. *destruens* AKL-3 on larval mortality and development period of *S*. *litura*.

Concentration (mg/ml)	Larval mortality (%)	Larval period (days)	Pupal period (days)	Total development period (days)
Control	03.33 ± 03.72^a^	13.08 ± 0.17^a^	8.73 ± 0.22^a^	21.81 ± 0.25^a^
0.5	10.00 ± 04.56^a^	13.61 ± 0.29^a^	8.82 ± 0.27^a^	22.62 ± 0.35^ab^
1.0	30.00 ± 05.56^ab^	13.84 ± 0.25^a^	10.00 ± 0.35^ab^	24.40 ± 0.98^bc^
1.5	33.33 ± 10.21^ab^	13.39 ± 0.45^a^	10.96 ± 0.35^ab^	25.43 ± 0.84^c^
2.0	53.33 ± 10.86^bc^	16.72 ± 0.46^b^	12.40 ± 0.27^b^	29.70 ± 0.49^d^
2.5	70.00 ± 06.97^c^	17.63 ± 0.29^b^	13.00 ± 0.0^b^	31.00 ± 0.00^d^
F	13.55**	41.92**	3.45*	50.00**

Values are Means ± S.E. Means followed by different superscript letters within a column are significantly different. Tukey’s test p ≤ 0.05. **Significant at 1%, *Significant at 5%.

### Sub lethal effects

**Figure 5:**
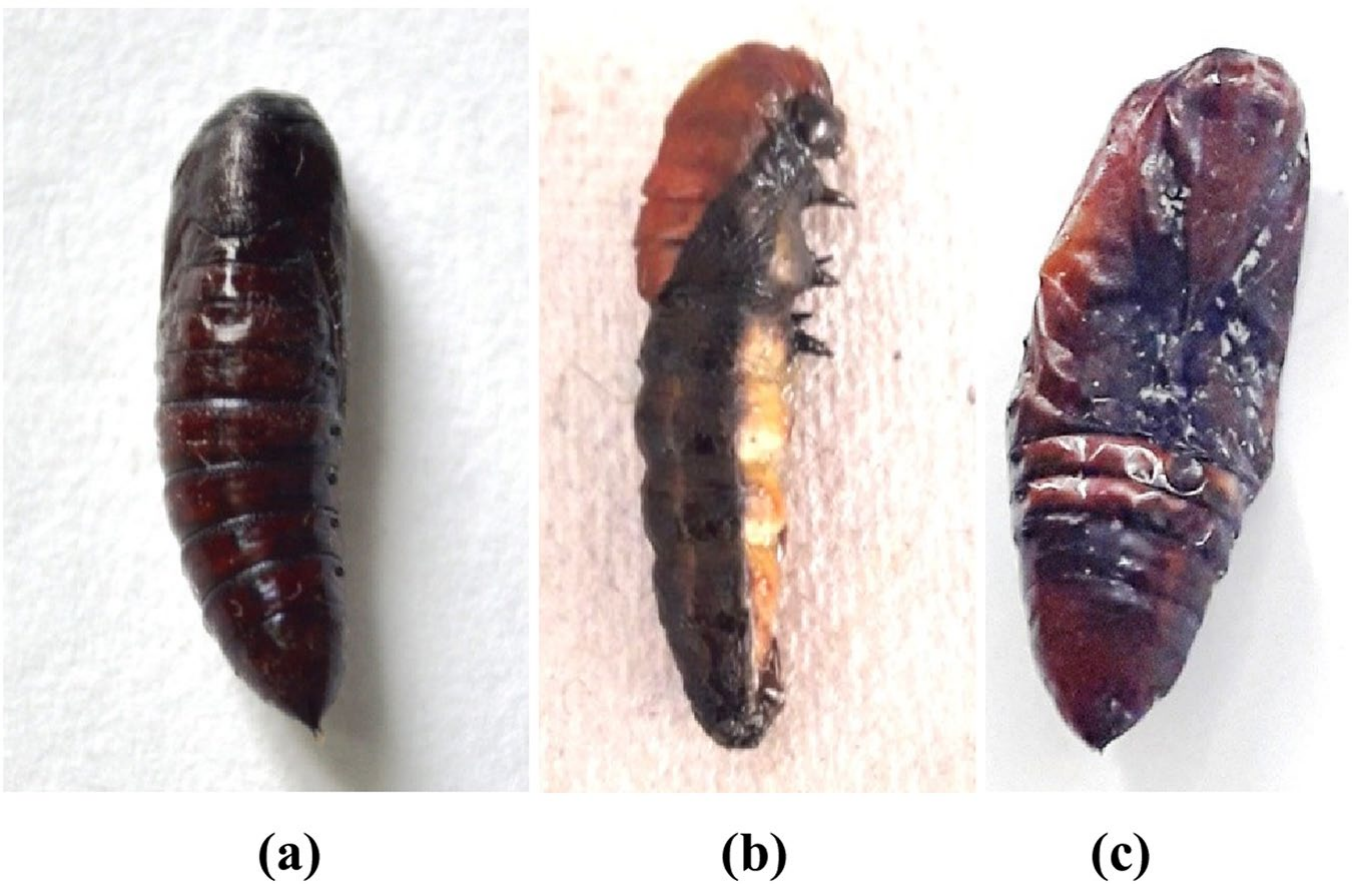
Effect of α-glycosidase inhibitors from *A*. *destruens* AKL-3 on pupae of *S*. *litura* (**a**) normal, (**b**) larval pupal intermediate, (**c**) deformed.

**Figure 6:**
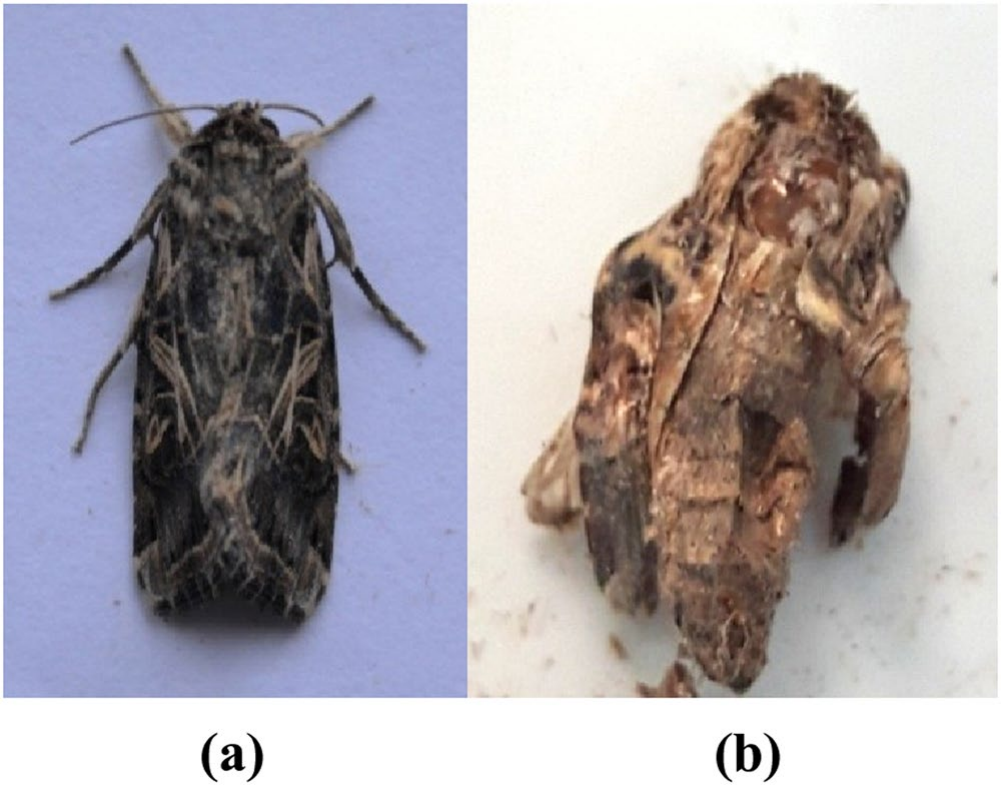
Effect of α-glycosidase inhibitors from *A*. *destruens* AKL-3 on adults of *S*. *litura* (**a**) normal, (**b**) deformed.

Sub lethal effects in the form of morphological deformities like larval pupal intermediates as well as pupae with attached larval exuviae, depressed head and unsclerotised cuticle were also manifested in *S*. *litura* (Fig. 5a–c). However, in some of the cases pupae were normal but the adults emerged with underdeveloped and crumpled wings (Fig. 6a,b).

### Nutritional physiology

Nutritional analysis revealed a significant influence of pooled fraction of *A*. *destruens* AKL-3 culture on food utilization efficiency of *S*. *litura* (Table 2). A significant decline of 31.96–53.94 percent in relative growth rate (RGR) over control was recorded when fed on diet amended with different concentrations of the pooled fraction (F = 155.43, p ≤ 0.001). It was observed to be a dose dependent effect. Similarly, a significant decrease was recorded in relative consumption rate (RCR), which dropped by 19.24–72.93 percent over control at different concentrations (F = 442.51, p ≤ 0.001). The highest concentration of the inhibitor was found to be the most effective. Similar deleterious effects were observed in the efficiency of conversion of ingested (ECI) and digested food (ECD) of *S*. *litura* (ECI: F = 115.91, p ≤ 0.001; ECD: F = 255.66, p ≤ 0.001). The highest concentration exhibited maximum inhibitory effect by dropping the ECD by 2.85 times over control.

**Table 2:** Effect of α-glycosidase inhibitors from *A*. *destruens* AKL-3 on nutritional physiology of *S*. *litura larvae*.

Concentration (mg/ml)	RGR (mg/mg/day)	RCR (mg/mg/day)	ECI (%)	ECD (%)	AD (%)
Control	1.94 ± 0.02^a^	16.48 ± 0.35^a^	7.42 ± 0.15^a^	7.53 ± 0.16^a^	99.68 ± 0.19
0.5	1.32 ± 0.05^b^	13.31 ± 0.32^b^	5.96 ± 0.30^b^	6.09 ± 0.11^b^	99.64 ± 0.15
1.0	1.25 ± 0.02^b^	08.97 ± 0.18^c^	4.67 ± 0.18^c^	4.83 ± 0.11^c^	99.62 ± 0.02
1.5	1.12 ± 0.04^c^	07.42 ± 0.16^d^	3.99 ± 0.07^d^	4.09 ± 0.89^d^	99.44 ± 0.55
2.0	1.08 ± 0.03^c^	05.60 ± 2.18^e^	3.49 ± 0.04^de^	3.39 ± 0.09^e^	99.29 ± 0.36
2.5	0.89 ± 0.02^d^	04.45 ± 0.19^f^	3.06 ± 0.16^e^	2.64 ± 0.11^f^	99.23 ± 0.04
F	155.43**	442.51**	115.91**	255.66**	NS

RGR = Relative growth rate, RCR = Relative consumption rate, ECI = Efficiency of conversion of ingested food, ECD = Efficiency of conversion of digested food, AD = Approximate digestibility. Values are Mean ± SE. Means followed by different superscript letters within a column are significantly different (p ≤ 0.05) based on Tukey’s test. **Significant at 1%, NS non-significant.

### *In vivo* effects on digestive enzymes

Addition of inhibitory fraction of *A*. *destruens* AKL-3 to larval diet significantly decreased α-glucosidase activity in the insect gut. As compared to control, there was 11.49–47.74 percent decline in the level of α-glucosidase activity after 48 hr (F = 262.34, p ≤ 0.001; Table 3). The larvae feeding on the highest concentration of pooled fraction showed 48.67 percent reduction in enzyme activity after 72 hr over control (F = 368.64, p ≤ 0.001). Similar effects were recorded on β-glucosidase. The decrease in enzyme activity was observed in the range of 18.96–42.06 percent after 48 hr (F = 135.46, p ≤ 0.001; Table 4). Although prolonged exposure to pooled fraction did not further drop the level of enzyme activity, but in comparison to control it remained significantly lower (F = 29.53, p ≤ 0.001). Pooled fraction also significantly suppressed the level of α-amylase of *S*. *litura* larvae. Larval exposure to amended diet for 48 hr reduced the α-amylase activity by 18.57–25.37 percent at higher concentrations (F = 36.29, p ≤ 0.001; Table 5) and 14.57–30.42 percent after 72 hr in comparison to control larvae (F = 104.73, p ≤ 0.001).

**Table 3:** Effect of α-glycosidase inhibitors from *A*. *destruens* AKL-3 on α-glucosidase activity of *S*. *litura* larvae.

α-Glucosidase activity (µmol/mg)		
Concentration (mg/ml)	48 hours	72 hours
Control	32.89 ± 1.09^a^	40.35 ± 0.25^a^
0.5	29.10 ± 0.42^b^	38.54 ± 1.11^a^
1.0	22.03 ± 0.43^c^	34.62 ± 0.16^b^
1.5	20.5 ± 0.08^cd^	31.18 ± 0.21^c^
2.0	19.26 ± 0.20^de^	29.16 ± 0.21^c^
2.5	17.18 ± 0.33^e^	20.71 ± 0.50^d^
F	262.34**	368.64**

Values are Mean ± SE. Means followed by different superscript letters within a column are significantly different (p ≤ 0.05) based on Tukey’s test. **Significant at 1%.

**Table 4:** Effect of α-glycosidase inhibitors from *A*. *destruens* AKL-3 on β-glucosidase activity of *S*. *litura* larvae.

β-Glucosidase activity (µmol/mg)		
Concentration (mg/ml)	48 hours	72 hours
Control	37.28 ± 0.69^a^	51.97 ± 2.16^a^
0.5	30.21 ± 0.97^b^	48.60 ± 2.54^ab^
1.0	28.86 ± 0.52^bc^	43.10 ± 0.28^bc^
1.5	26.65 ± 0.06^cd^	42.41 ± 0.07^c^
2.0	24.52 ± 0.27^d^	39.55 ± 0.19^c^
2.5	21.60 ± 0.91^e^	37.80 ± 0.79^c^
F	135.46**	29.53**

Values are Mean ± SE. Means followed by different superscript letters within a column are significantly different (p ≤ 0.05) based on Tukey’s test. **Significant at 1%.

**Table 5:** Effect of α-glycosidase inhibitors from *A*. *destruens* AKL-3 on α-amylase activity of *S*. *litura* larvae.

α-Amylase activity (µmol/mg)		
Concentration (mg/ml)	48 hours	72 hours
Control	79.57 ± 0.86^a^	88.90 ± 1.41^a^
0.5	75.47 ± 2.91^ab^	75.95 ± 2.31^b^
1.0	74.11 ± 1.12^ab^	70.33 ± 1.38^c^
1.5	70.93 ± 0.79^bc^	66.39 ± 0.52^cd^
2.0	64.81 ± 1.71^cd^	64.16 ± 0.92^d^
2.5	59.38 ± 2.05^d^	61.86 ± 0.97^d^
F	36.29**	104.73**

Values are Mean ± SE. Means followed by different superscript letters within a column are significantly different (p ≤ 0.05) based on Tukey’s test. **Significant at 1%.

### Antifungal activity

**Figure 7:**
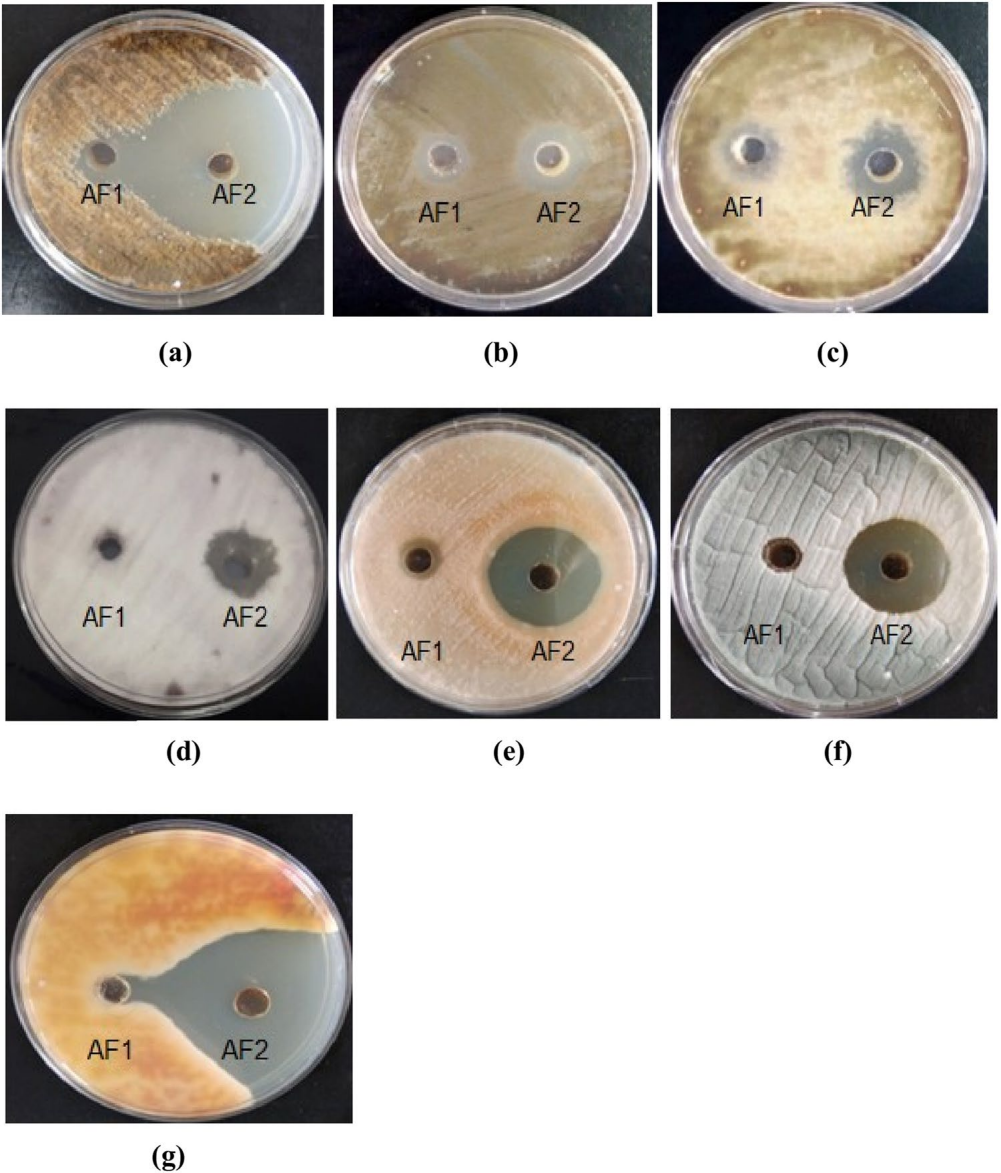
Zones of inhibition exhibited by active fractions AF1 and AF2 of *A*. *destruens* AKL-3 against different phytopathogenic fungi (**a**) *A*. *brassicicola* (**b**) *A*. *alternata* (**c**) *A*. *mali* (**d**) *C*. *beticola* (**e**) *F*. *oxysporum* (**f**) *C*. *herbarum* (**g**) *C*. *gloeosporioides*.

**Figure 8:**
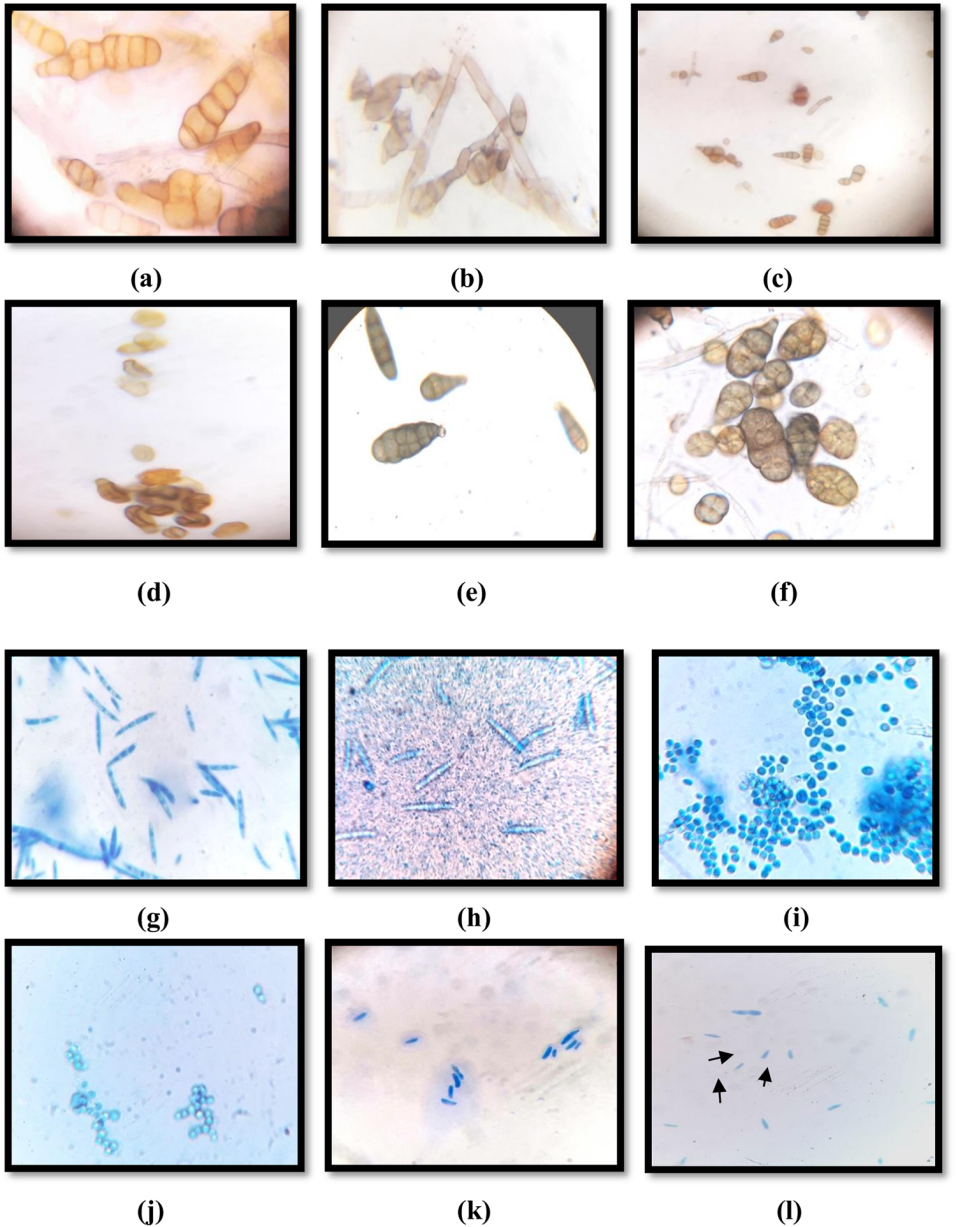
Effect of active fraction AF2 of *A*. *destruens* AKL-3 on mycelial structure and spores of *A*. *brassicicola* (**a**: untreated, **b**: treated), *A*. *alternata* (**c**: untreated, **d**: treated), *A*. *mali* (**e**: untreated, **f**: treated), *C*. *beticola* (**g**: untreated, **h**: treated), *C*. *herbarum* (**i**: untreated, **j**: treated), *F*. *oxysporum* (**k**: untreated, **l**: treated).

Both the active fractions of *A*. *destruens* AKL-3 were examined for their antifungal activity against phytopathogens. It was observed that active fraction AF2 was more potent in terms of its antifungal potential. Even at a lower concentration (250 µg/ml) it evinced higher antifungal activity as compared to AF1 (500 µg/ml). The fraction AF1 exhibited antagonist effect only against all tested *Alternaria spp*. and *Cercospora beticola* Sacc., producing inhibitory zones in the range of 10–11 mm whereas AF2 inhibited all the test pathogens producing higher zones of inhibition (22–44 mm) (Fig. 7a–g). The results of antifungal activity of AF2 prompted the examination of the spores and mycelial structures of the affected phytopathogens *viz*. *Alternaria brassicicola* (Schwein.) Wiltshire, *Alternaria mali* Roberts., *Alternaria alternata* (Fr.) Keissl., *C*. *beticola*, *Cladosporium herbarum* (Pers.) Link, *Colletotrichum gloeosporioides* (Penz.) Sacc. and *Fusarium oxysporum* Schltdl.. Light microscopic studies demonstrated severe morphological abnormalities such as leakage of cellular material, thinning of hyphae, formation of vesicles, discoloration of hyphae and alteration in the spore morphology caused by metabolites near the inhibition zone. In *A*. *brassicicola* pronounced effects as manifested in mycelial breakage, deformed and shrunken spores and hyphal swellings resulting in bulbous structures were seen under light microscope (Fig. 8a,b). Shrunken and distorted spores were also observed in *A*. *alternata* and *A*. *mali* (Fig. 8c–f). No visible effects on hyphae were observed. In *C*. *beticola* and *C*. *herbarum* leakage of cytoplasmic content as well as morphological deformities were observed in spores as noticed in *A*. *brassicicola*. The treated spores were lightly stained as compared to untreated ones (Fig. 8g–j). In *F*. *oxysporum* reduction in the size of spores was observed (Fig. 8k,l).

## Discussion

In the present study endophytes have been isolated from *C*. *gigantea* and screened for their α-glycosidase (α-glucosidase and α-amylase) inhibitory potential. Maximum α-glucosidase inhibitory potential was demonstrated by a culture AKL-3, identified to be *Alternaria destruens*. Although, a number of reports are available on endophytes with ability to produce α-glycosidase inhibitors^37–42^, this is the first study reporting the α-glycosidase inhibitory potential of an endophytic *A*. *destruens*. The isolate also exhibited good insecticidal and antifungal activity. *A*. *destruens* is a dothideomycetous fungus patented as a bioherbicide for controlling dodder species, a serious parasitic weed in the crops^43^, but no reports are available on its bio-control ability against insect pests and pathogens. Inhibitors of α-glycosidases have been recognized as inherent mechanisms of defense against insect pests^23–25^, but only a few reports are available on the insecticidal potential of digestive enzyme inhibitors from endophytic fungi^40,42^. Detrimental effects of α-glucosidase inhibitors produced by an endophytic *Exophiala spinifera* (Nielsen & Conant) McGinnis on *S*. *litura* have been documented by Kaur *et al*.^42^. In this study, partially purified AGIs from *A*. *destruens* were biochemically characterized as phenolic compounds after staining positively with Fast blue B and FeCl_3_. High larval mortality induced in the presence of inhibitory fraction could be attributed to phenolic nature of α-glycosidase inhibitory compounds. Detrimental effects of phenolic compounds on insect pests have been documented by Singh *et al*.^44^ and Singh *et al*.^45^. The AGI’s of *A*. *destruens* AKL-3 also delayed the overall development period. Delay in the development period of *S*. *litura* when fed on diet supplemented with phenolic compounds has been reported by other workers^45,46^. The molting process was also affected under the influence of *A*. *destruens* inhibitors, which caused morphological deformities like larval-pupal intermediates, undeveloped pupae and adults with underdeveloped and crumpled wings. It is possible that the AGIs obtained in the present study could be affecting the chitinase enzymes involved in the process of molting. Inhibitors of chitinase are known for their insecticidal activity^47^. Effect of inhibitors from *A*. *destruens* AKL-3 was also observed on nutritional analysis as it affected all the nutritional indices. Relative consumption and growth rate of larvae feeding on diet containing different concentrations of inhibitory compounds of *A*. *destruens* AKL-3 was also significantly reduced as compared to control. As consumption rate is directly proportional to growth rate, low consumption rate indicates antifeedant effects of the inhibitor. A decreased value of ECI indicated that food ingested was being mainly utilized for energy required for detoxification and less was being metabolized to insect biomass. With the increased requirement for energy, major proportion of digested food is utilized to fulfill energy requirement, hence lowering the ECD value. This diversion of energy from biomass production into detoxification reduces the growth^48^. Also, i*n vivo* evaluation on *S*. *litura*’s digestive enzymes was carried out to determine whether the deleterious effects being observed under the influence of the inhibitors could be mediated by inhibiting digestive enzymes of insect. The *in vivo* studies corroborated the *in vitro* results and activity of all the tested digestive enzymes of *S*. *litura* was lowered to a significant level in *in vivo* studies. The inhibitory effects of phenolics on digestive enzymes have been reported in literature. Singh *et al*.^45^ reported the detrimental effects of chlorogenic acid isolated from an endophytic *Cladosporium velox* Zalar, de Hoog & Gunde-Cim., on digestive enzymes of *S*. *litura*. Karthik *et al*.^49^ isolated phenolics from the lichen *Heterodermia leucomela* (L.) Poelt (Caliciales: Physciaceae) and found them to inhibit digestive enzymes of mosquito *Aedes aegypti* L. (Diptera: Culicidae). Campbell *et al*.^50^ documented the inhibitory effects of an AGI castanospermine on disaccharide enzymes of sap feeding insects.

Glycosidase inhibitors have also been reported to possess antifungal activities^51^. Microscopic observations of phytopathogenic fungi treated with active fraction AF2 revealed severe detrimental effects on mycelium as well as on fungal spores. It is possible that inhibitory metabolites attack the cell wall as indicated by morphological alterations in vegetative cells and spores. As previously suggested they could be inhibiting the enzyme chitinase which is involved in processes during fungal growth and has a role in synthesis and extension of cell wall^30^. Therefore, glycosidase inhibitors can induce antifungal effects by affecting the chitinase enzymes^52^. In *in vitro* studies, it was determined that AF2 also possessed α-amylase and β-glucosidase inhibitory activities, which may be the contributing factors in increasing its antifungal activity. Kim *et al*.^53^ reported the antifungal activity of a β-glucosidase inhibitor which affected the hydrolytic enzymes of fungi.

## Conclusion

This is the first report of α-glycosidase inhibitors from endophytic *A*. *destruens*. The study reveals the potential of endophytic fungi as sources of α-glycosidase inhibitors with insecticidal and antifungal potential. The present study also demonstrates that α-glycosidase inhibitory potential can be used as strategy for screening of endophytes with bio-control potential against pests and pathogens.

## Materials and Methods

### Isolation of endophytic fungi

Endophytic fungi were isolated from different parts *viz*. leaves and stems of healthy *C*. *gigantea* plants. After washing with running tap water, the plant parts were thoroughly rinsed with distilled water. Surface sterilization was carried out with 70% ethanol for 1–2 min followed by treatment with 4% sodium hypochlorite solution for 2–3 min. Again the plant parts were rinsed with sterilized distilled water. To ensure surface sterilization, the water obtained after last wash was plated on PDA. The plant parts (5–6 pieces) in the size range of 2–5 mm were inoculated on water agar plates supplemented with chloramphenicol (20 µg/ml) (HiMedia, Mumbai, India). Plates were incubated at 30 °C for 3–4 days to few weeks till the hyphae emerged. The emerging fungal hyphae from inoculated plant parts, were picked, purified and preserved on PDA slants for further studies^42^.

### Production of secondary metabolites

The production was carried out in Erlenmeyer flasks (250 ml) containing 50 ml of malt extract broth (dextrose 2%, malt extract 2%, protease peptone 0.1%, pH 5.5)^42^. The production medium was inoculated with one plug (8 mm diameter) taken from periphery of freshly grown purified culture. The flasks were incubated for 10 days on a rotary shaker at 250 rpm and 30 °C. After 10 days of incubation, 50 ml ethyl acetate was added to each of the flask and extraction was carried out at 120 rpm and 40 °C for 1.5 hr twice. The extracted organic phase was concentrated on rotary evaporator (BUCHI). The concentrated samples were re-suspended in HPLC grade water and used for further studies.

### Assay for α-glucosidase and β-glucosidase inhibition

Enzyme activity was determined in a microtiter 96-well plate. Reaction mixture consisting of 50 μl of phosphate buffer (50 mmol/l; pH 6.8), 10 μl of α-glucosidase enzyme (1U/ml) from *Saccharomyces sp*. (HiMedia) and 20 μl of inhibitory extract was pre-incubated at 37 °C for 5 min. After 5 min, 20 μl of 2 mmol/l pNPG substrate (HiMedia) (prepared in 50 mmol/l phosphate buffer, pH 6.8) was added followed by incubation at 37 °C for 30 min. Termination of reaction was carried out by addition of 50 μl of sodium carbonate (100 mmol/l)^42^. Acarbose was used as a positive control. The breakdown of pNPG into yellow coloured *p*-nitrophenol was quantified by reading the absorbance at 405 nm. Every experiment was performed in triplicate, along with appropriate blanks. The % inhibition was calculated using the formula:

$$ \% \,{\rm{inhibition}}=\frac{{\rm{absorbance}}\,{\rm{of}}\,{\rm{control}}\,\mbox{--}\,{\rm{absorbance}}\,{\rm{of}}\,{\rm{sample}}\times 100}{{\rm{absorbance}}\,{\rm{of}}\,{\rm{control}}}$$

For the determination of β-glucosidase inhibitory potential same procedure was applied using *p*-nitrophenyl–β-d-glucopyranoside as substrate.

### Assay for α-amylase inhibition

The assay was conducted as described by Nair *et al*.^54^ with slight modification. The assay mixture containing 200 μl of 20 mmol/l sodium phosphate buffer, 40 μl of α-amylase enzyme from porcine pancreas (2 U/ml) (HiMedia) and 40 μl of fungal extract was incubated for 10 min at 37 °C, followed by addition of 50 μl of starch in all test tubes and further incubated for 20 min. The reaction was terminated with the addition of 500 μl DNS reagent and placed in boiling water bath for 5 min, cooled and diluted with 5 ml of distilled water. Absorbance was measured at 540 nm. The control samples were prepared without any fungal extract. Acarbose was used as positive control.

### Identification of the producer culture

Selected culture was identified on the basis of morphological and molecular analysis. Morphological studies were conducted using slide culturing as described by Larone^55^. Thin layer PDA (potato dextrose agar) plates were prepared and small blocks were cut. These blocks were placed aseptically onto glass slide and inoculated with fungal culture. Coverslip was placed over it and incubated at 30 °C. The branching pattern, sporulation and arrangement of the hyphae were examined under microscope (OLYMPUS BX 60).

### Phylogenetic analysis

The culture AKL-3 was identified on molecular basis by amplification of ITS1-5.8S-ITS2 rDNA region using primer pair ITS1 and ITS4 by National Centre for Cell Science (NCCS), Pune (Maharashtra), India. Phylogenetic analysis of AKL-3 culture was conducted by NCCS, Pune.

### Partial purification and biochemical analysis

For partial purification extract was loaded onto a silica gel (100–200 mesh size) column (2 × 25 cm). The solvent system used was chloroform: ethyl acetate: formic acid in 5:4:1 ratio. Using this solvent system, fractions of 10 ml each were collected and activity was observed in fraction no. 11 and 14 which were designated as AF1 and AF2, respectively. Chemical nature of active fractions was determined using various TLC based biochemical methods using different visualization reagents *viz*. Dragendroff’s reagent for alkaloids, FeCl_3_ and Fast Blue B for phenols, ninhydrin for amine group, *p*-anisaldehyde for the detection of steroids and terpenoids.

### Insecticidal activity

#### Insect culture

Insecticidal activity was determined on larvae of *S*. *litura*. The culture was collected from fields around Amritsar (Punjab), India. It was reared on *Ricinus communis* L. (Euphorbiaceae) leaves in glass jars (15 × 10 cm) at 25 ± 2 °C and 65 ± 5% relative humidity in the laboratory. Hygienic conditions were maintained by changing leaves regularly until pupation. The emerging pupae were separated and kept in pupation jars (15 × 10 cm) with 2–3 cm layer of moist sterilized sand covered with filter paper. The adult moths on emergence were transferred to oviposition jars in the ratio of (1:2) males and females|. For nourishment of adults, cotton swab dipped in water and honey solution (4:1) was provided daily as food. To facilitate egg laying the oviposition jars were lined with filter paper. On hatching larvae were maintained on artificial diet as recommended by Koul *et al*.^56^ with slight modifications.

#### Bioassay studies

Insecticidal potential of both active fractions (AF1 and AF2) of *A*. *destruens* AKL-3 was evaluated individually as well as after pooling them together, on *S*. *litura* at a concentration of 1.5 mg/ml. Pooled fraction of *A*. *destruens* AKL-3 induced significantly higher mortality than individual fractions. Therefore, detailed studies on various parameters *viz*. larval mortality, total development period were conducted using different concentrations (0.5, 1.0, 1.5, 2.0, 2.5 mg/ml) of pooled fraction. Diet without inhibitor was taken as control. The experiment was designed randomly with six treatments including control and five replications per treatment. Six second instar larvae (6 days old) were taken per replication. Plastic containers (4 × 6 cm) were used to rear larvae individually on treated and control diets. The experiment was conducted under controlled temperature and humidity condition of 25 ± 2 °C and 65 ± 5%, respectively^42^. The diet was changed regularly on alternate days and larvae were checked daily for survival. Observations were made on larval mortality, larval period, pupal period as well as adult emergence. Furthermore, observations were recorded on morphological deformities in larvae, pupae and adults.

#### Nutritional analysis

The gravimetric method given by Waldbauer^57^ was used to determine the nutritional indices. For each concentration of inhibitor, twenty five second instar (6 days old) larvae were starved for 3–4 hr and fed on artificial diet amended with 0.5–2.5 mg/ml of inhibitor from *A*. *destruens* and unamended diet served as control. Larvae were maintained in plastic containers (4 × 6 cm) containing a known amount of diet. Optimum temperature and humidity of 25 ± 2 °C, and 65 ± 5%, respectively were maintained. After termination of experiment i.e. after 72 hr larvae, residual diet and faecal matter were separated, dried by incubation at 60 °C for 72 hr and weighed. All nutritional indices were calculated using dry weights and therefore 25 second instar larvae and 25 diet samples were dried to a constant weight to determine fresh/dry weight ratios^58^. Nutritional indices were calculated as per Wheelar and Isman^59^ by using following formulae:

$$\begin{array}{rcl}{\rm{RGR}} & = & \frac{{\rm{Change}}\,{\rm{in}}\,{\rm{larval}}\,{\rm{dry}}\,\mathrm{weight}/\mathrm{day}}{{\rm{Initial}}\,{\rm{larval}}\,{\rm{dry}}\,{\rm{weight}}}\\ {\rm{RCR}} & = & \frac{{\rm{Change}}\,{\rm{in}}\,{\rm{diet}}\,{\rm{dry}}\,\mathrm{weight}/\mathrm{day}}{{\rm{Initial}}\,{\rm{larval}}\,{\rm{dry}}\,{\rm{weight}}}\\ {\rm{ECI}} & = & \frac{{\rm{Dry}}\,{\rm{weight}}\,{\rm{gain}}\,{\rm{of}}\,{\rm{insect}}}{{\rm{Dry}}\,{\rm{weight}}\,{\rm{of}}\,{\rm{food}}\,{\rm{ingested}}}\times 100\\ {\rm{ECD}} & = & \frac{{\rm{Dry}}\,{\rm{weight}}\,{\rm{gain}}\,{\rm{of}}\,{\rm{insect}}}{{\rm{Dry}}\,{\rm{weight}}\,{\rm{of}}\,{\rm{food}}\,{\rm{ingested}}-{\rm{Dry}}\,{\rm{weight}}\,{\rm{of}}\,{\rm{frass}}}\times 100\\ {\rm{AD}} & = & \frac{{\rm{Dry}}\,{\rm{weight}}\,{\rm{of}}\,{\rm{food}}\,{\rm{ingested}}-{\rm{Dry}}\,{\rm{weight}}\,{\rm{of}}\,{\rm{frass}}}{{\rm{Dry}}\,{\rm{weight}}\,{\rm{of}}\,{\rm{food}}\,{\rm{ingested}}}\times 100\end{array}$$

RGR = Relative growth rate, RCR = Relative consumption rate, ECI = Efficiency of conversion of ingested food, ECD = Efficiency of conversion of digested food, AD = Approximate digestibility, d = day.

### Effect on glycosidase enzymes of *S*. *litura*

To determine the activity of α-glucosidases, β-glucosidases and α-amylase *in vivo*, late second instar larvae were fed on a diet supplemented with different concentrations ranging from 0.5–2.5 mg/ml of the inhibitor as well as control diet for 48 hr and 72 hr. Ten larvae per replication were used for each time interval and the experiment was replicated thrice. Homogenates (1% w/v) were used as extracts and prepared by homogenizing larval midguts (25 mg) in 2.5 ml distilled water followed by transfer to 1.5 ml centrifuge tubes and centrifuged at 13000 g for 20 min at 4 °C. α-Amylase and α-, β-glucosidase enzyme activities of homogenates were then assayed^42^.

### Evaluation of antifungal activity

Antifungal activity was determined against various phytopathogens *viz*. *A*. *brassicicola* (MTCC 2102), *A*. *mali* (lab isolate), *A*. *alternata* (lab isolate), *C*. *gloeosporioides* (lab isolate), *C*. *beticola* (KJ461435), *C*. *herbarum* (MTCC 351), *F*. *oxysporum* (MTCC 284). Test fungi were inoculated on PDA plates and punctured with sterile cork borer to make wells (6 mm diameter). The inhibitor (200 µl) was transferred to each well under aseptic conditions and incubated at 30 °C for three days. The concentrations of inhibitors used were 250 µg/ml for AF2, 500 µg/ml for AF1. The antifungal activity was observed as clear zones of inhibition around wells and measured in millimeters (mm). The deformed hyphae of tested phytopathogens at zones of inhibition were picked and stained with lactophenol blue dye and examined under microscope for detection of morphological changes.

### Statistical analysis

Each experiment was performed in triplicate except for bioassay studies were six replicates were taken. To compare difference in means, one way analysis of variance (ANOVA) with Tukey’s test at P ≤ 0.05 was performed. SPSS v17.0 software for windows version and Microsoft office excel 2007 (Microsoft Corp., USA) were used to perform the statistical analysis. The data on larval mortality was subjected to probit analysis for calculation of LC_50_ value.

### Ethical approval and consent to participate

This article does not contain any studies involving human participants or animals performed by any of the authors.

## Data Availability

All data generated or analyzed during this study are included in this published article.
